# Army Meteorological Register for Twelve Years, from 1831 to 1842, Inclusive

**Published:** 1852-01

**Authors:** 


*Army Meteorological Register for Twelve Years, from* 1831 *to* 1842, *inclusive.*

For the last thirty years meteorological observations, embracing facts pertaining to temperature, weight of the atmosphere, winds, rain, and the dew point, have entered into the daily duties of medical officers of the army stationed at the different military posts throughout the country. The work, the title page of which is given above, contains a collection of these observations for a period of twelve years. No attempt to deduce results, from the mass of data accumulated, is made. The facts alone are presented "to those interested in the progress of meteorology, trusting that, in the hands of such as enjoy leisure, opportunity, and a love of the subject, they may be profitably employed in the elucidation of many of the interesting theories of this important science."

Meteorological generalizations are of importance in connection with the subject of ætiology and the fluctuations incident to the character of diseases at different times and places. Here is a vast uncultivated field for analytical researches. The Army Meteorological Register will be found highly useful in such an investigation. Much might be said to incite the ambitious votary of science to explore this territory, but our limits forbid amplification. The Army Register, in its present shape, is like the quarry, which, by means of labor and art, furnishes the polished marble.

